# Supplementary material for: Common Structural Pattern for Flecainide Binding in Atrial-Selective Kv1.5 and Nav1.5 Channels: A Computational Approach
Source: Pharmaceutics. 2022 Jun 27;14(7):1356. doi: 10.3390/pharmaceutics14071356 (PMC9318806; doi:10.3390/pharmaceutics14071356)
Supplement: Supplementary file 1 [file pharmaceutics-14-01356-s001.zip › pharmaceutics-1748843-supplementary.pdf]

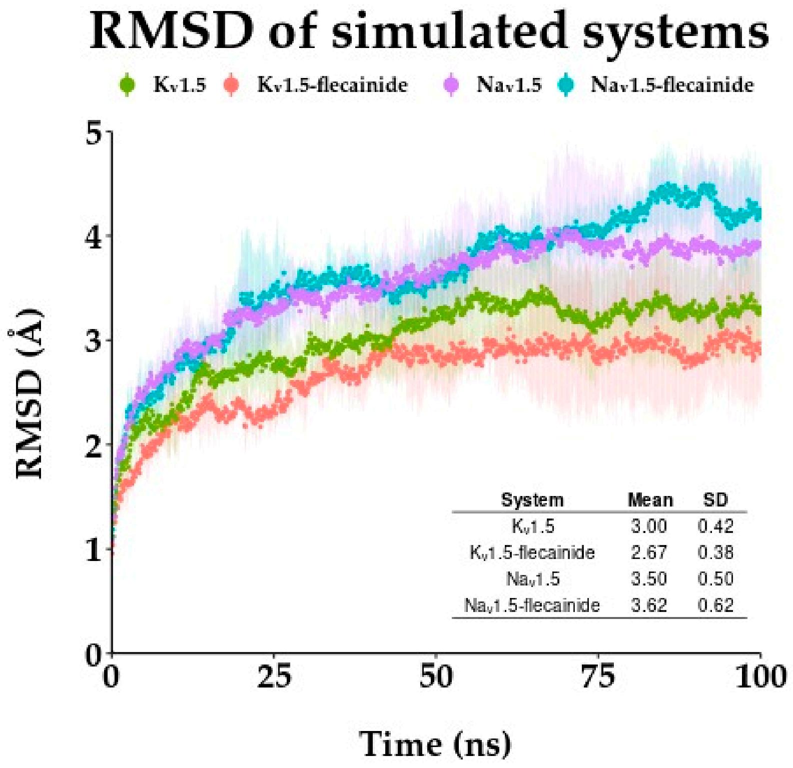

Figure S1. Root-mean square deviation (RMSD) of proteins atoms. The RMSD average values (colored points) from the three molecular dynamics (MD) replicas of $K_v1.5$ and $Na_v1.5$ apo and holo systems (flecainide-bound) are displayed. Verticals lines represent the RMSD standard deviation. RMSD values were computed regarding the first frame of each MD. *SD: standard deviation.*

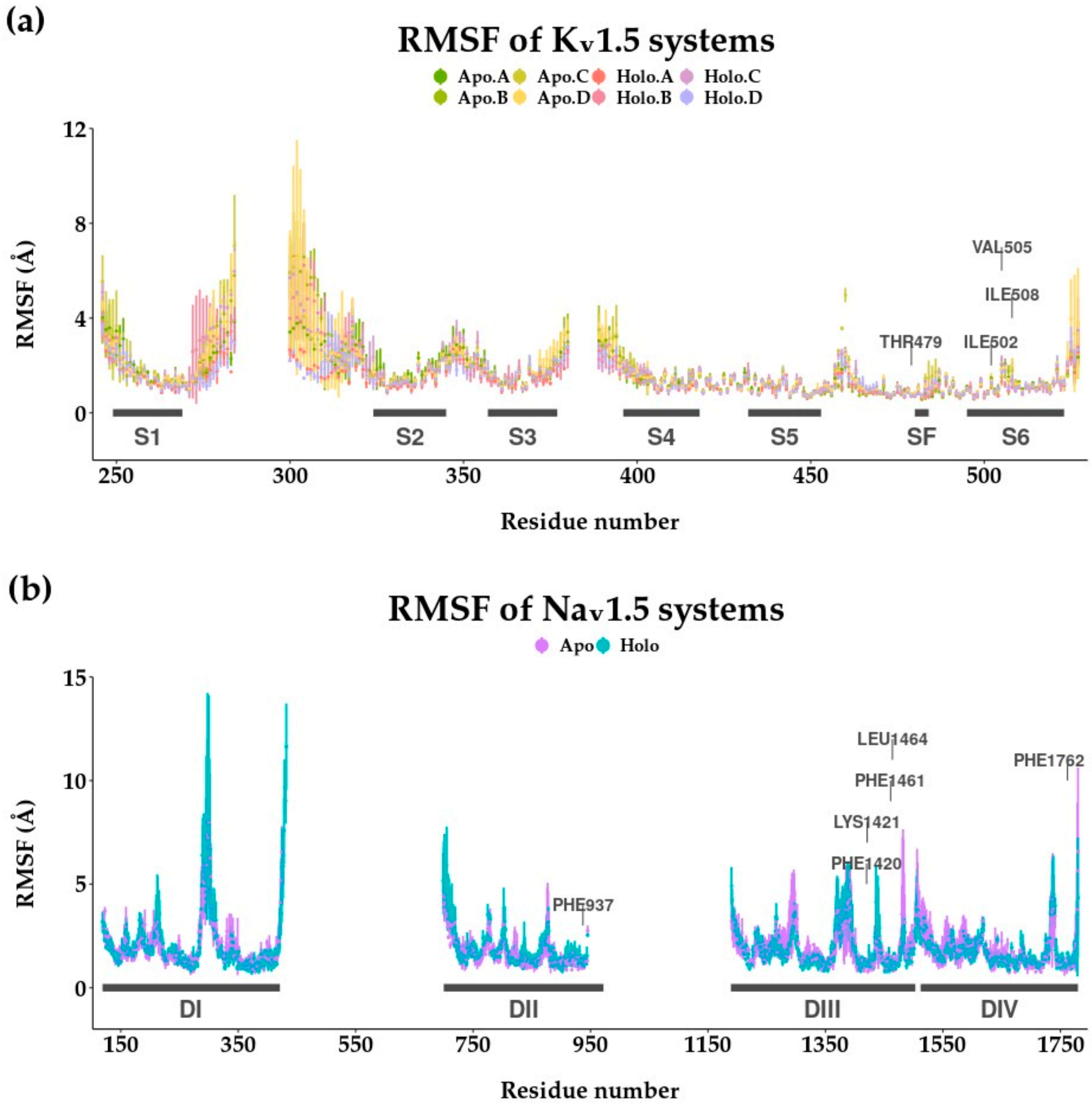

Figure S2. The Root Mean Square Fluctuation (RMSF) of residue atoms. Averages of RMSF (colored points) from the three MD replicas, comparing with the first frame, are shown in apo and holo systems. Vertical lines represent the RMSF standard deviation. The residues relevant for flecainide bindings as reported by mutagenesis in K_v1.5 and cryo-EM Na_v1.5-flecainide structure are indicated [22,23]. (**a**) For K_v1.5, the regions corresponding to transmembrane segments S1-S6 for each A, B, C and D subunits are indicated. Missing RMSF values correspond to gap regions in K_v1.5 model (residues from loop S1-S2 and loop S3-S4) (**b**) For Na_v1.5, domains DI-DIV are labeled. The gap corresponds to the missing residues from linkers between domains DI-DII and DII-DIII.

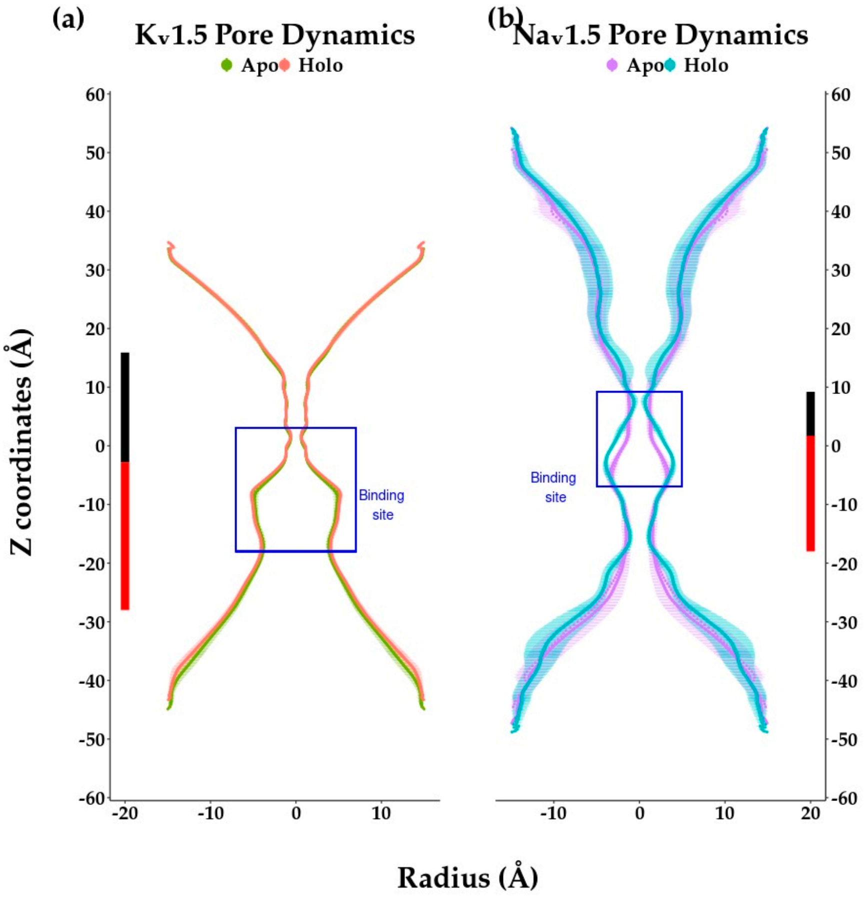

Figure S3. Radius profile of ion channel pore. The average radius value (colored point) from the three MDs replicas along z-axis is displayed for (**a**) K_v1.5 and (**b**) Na_v1.5 in apo and holo systems. Horizontal lines represent the radius standard deviation. Black and red lines stand for selectivity filter and central cavity, respectively.
